# Development of a Cervical Cancer Screening Program in Rural Guatemala

**DOI:** 10.9745/GHSP-D-24-00282

**Published:** 2025-08-14

**Authors:** Taryn McGinn Valley, Elizabeth White, Alli Foreman, Alejandro Chavez, Tana Chongsuwat, Linda Foxworthy, Madhuri Reddy, Cecilia Arroyave, Kevin Wyne, Rafael Tun, Yoselin Emelina Letona López, Dominga Pic Salazar, Cesia Castro Chutá, Sean Duffy

**Affiliations:** a University of Wisconsin-Madison, Madison, WI, USA.; b University of California-San Diego, San Diego, CA, USA.; c University of California-San Francisco, San Francisco, CA, USA.; d Charles River Community Health, Brighton, MA, USA.; e University of Kansas, Kansas City, KS, USA.; f Liga Contra el Cáncer, Bogotá, Colombia.; g Hospital Obras Sociales Monseñor Gregorio Schaffer, San Lucas Tolimán, Sololá, Guatemala.

## Abstract

**Background:**

In San Lucas Tolimán (SLT), Guatemala, a rural municipality with a large Indigenous population, women seeking cervical cancer screening face many barriers. We describe the process from design to implementation of a culturally appropriate and accessible cervical cancer screening and treatment pilot program for women aged 30–49 years.

**Methods:**

After conducting a community needs assessment, we trained community health workers (CHWs) on basic cervical cancer pathophysiology and human papillomavirus (HPV) self-swab kit use. CHWs provided educational seminars and enrolled interested, eligible women in a mobile health application. Women collected samples at home and returned completed kits to CHWs, who sent the kits to a partner lab. Women who were positive for HPV received follow-up care at the local hospital, where physicians had received training in visual inspection with acetic acid (VIA) with same-day cryotherapy or thermocoagulation. Women with advanced lesions received access to care from gynecologists free of cost.

**Results:**

Between February and November 2023, of the 230 women eligible to participate in the program, 132 completed HPV self-swabs and received results, and 34 received positive HPV tests (25.76% prevalence). Sixty-seven women had VIA exams as their first screening. Women who received VIA exams had an overall positivity rate of 24.47% (23/94). Twenty-three women received treatment: cryotherapy (n=8), thermocoagulation (n=7), or loop electrosurgical excision procedure (n=8). SLT had higher HPV-positivity rates than nearby Escuintla (21.6%; *P*=.29) and significantly higher than Santiago Atitlán (17.4% HPV+; *P*=.02).

**Conclusion:**

Our screening program found significantly higher HPV-positivity rates in SLT than in previous Guatemalan studies. Our research reinforces that adequately treating cervical cancer in Guatemala requires accompaniment during care and economic support to make care affordable or free. Based on our pilot program, organizations worldwide can further invest in culturally sensitive cervical cancer screening and treatment.

## Abstract (precis)

We developed a cervical cancer screening and treatment pilot program led by community health workers to improve access for Indigenous women in rural Guatemala.

## Teaser

**Key Findings:**

- As cervical cancer continues to kill worldwide despite being preventable and treatable, human papillomavirus rates (HPV) are higher in San Lucas Tolimán, a rural, Indigenous municipality in highlands Guatemala, than previously documented elsewhere in the country and region.
- We showed the feasibility of a collaborative, community-based project providing wrap-around cervical cancer screening with HPV testing, local and affordable treatment, and high-quality follow-up.

**Key Implication:**

- Health care systems worldwide, both public and private, can use our model to further invest in culturally sensitive cervical cancer screening and treatment.

See supplementary material for the full-text article in Spanish.

## INTRODUCTION

Cervical cancer is extremely preventable and treatable; most cases are caused by unresolved human papillomavirus (HPV) infection.^1^ The World Health Organization (WHO) estimates that the worldwide prevalence of HPV among women is 11.7%, with estimates in Latin America around 16%.^2^^,^^3^ Death from cervical cancer is highly preventable due to HPV vaccines, various cervical cancer screening methods, and treatment of precancerous and cancerous lesions. Although the WHO has declared cervical cancer elimination feasible, hundreds of thousands of individuals continue to contract HPV and die from cervical cancer every year.^4^^,^^5^ Large-scale HPV vaccination campaigns and screening programs have effectively reduced the cervical cancer burden in high-income countries; however, HPV infections continue to rise in low- and middle-income countries (LMICs), where 80% of cervical cancer deaths occur.^4^

Scholars attribute the disproportionate cervical cancer incidence and mortality in LMICs, such as Guatemala, to international inequity in health care financing and resources, relatively resource-intensive screening, systemic inequitable barriers to care, and fragmented health care systems.^6^^–^^8^ Existing cervical cancer screening programs face compounding systemic and sociocultural challenges. Due to a lack of screening coverage, laboratory infrastructure, and staff, cervical cancer screening programs are less effective in LMICs.^9^^–^^11^ In historically disinvested health care systems, lack of follow-up care and electronic health record (EHR) systems worsen access to reliable or quality care.^6^^,^^8^^,^^12^ Sociocultural inequities also affect women’s ability and willingness to be screened.^6^^,^^13^^–^^16^ Women with more education and higher family incomes may be able to access information and exercise autonomy to be screened;^14^ women who experience ongoing violence may struggle to overcome the lack of information, logistics, and cost to receive care.^6^^,^^16^^,^^17^ Overall, cervical cancer screening programs in LMICs tend to be ineffective, resulting in continued high cervical cancer incidence and mortality.

In Guatemala, cervical cancer rates are among the highest in the Latin American and Caribbean region; the total burden of cervical cancer is estimated to be 22.3 cases per 100,000 women, with an HPV infection rate between 12.4% and 33%, depending on study location and methodology.^4^^,^^5^^,^^18^ Cervical cancer is the most common type of cancer and causes the most deaths among all cancer types for women of reproductive age in Guatemala.^19^

Guatemala’s nationalized health care service began an HPV vaccination campaign in 2017.^20^^,^^21^ The 2014–2024 National Plan for Prevention, Control, and Management of Cervical Cancer provided vaccines to girls aged 10–14 years.^22^ As of 2023, 85% of girls born between 2007 and 2009 had received at least 1 dose of the HPV vaccine, with lower rates for other birth years and receipt of the complete 2-dose vaccination.^10^^,^^23^ The Guatemalan government released a plan in 2023 to strengthen its cervical cancer vaccine program from 2024–2030,^10^ following the Pan American Health Organization and WHO plans to eliminate cervical cancer by 2030.

In terms of secondary cervical cancer prevention in Guatemala, scholars note institutional barriers, including unreliable results from laboratories and longstanding issues with health care access writ large.^24^ Overall, less than 40% of women in Guatemala have ever been screened for cervical cancer; screening rates are lower for Indigenous women living in rural areas.^10^^,^^18^ Therefore, most adult women in Guatemala may be at risk of developing cervical cancer.

Less than 40% of women in Guatemala have ever been screened for cervical cancer; screening rates are lower for Indigenous women living in rural areas.

Increasing regular screening is a cost-effective and recommended way to detect and treat precancerous and cancerous lesions, and scholars and policymakers have long discussed which cervical cancer screening techniques would most benefit Guatemala’s population. Debate continues as to whether there is one best screening modality for Guatemala overall, considering factors such as cost and scalability.^5^^,^^6^^,^^13^^,^^16^^,^^18^^,^^24^^–^^28^

At the time of our program design, the 3 modalities used in Guatemala were cervical cytology (Pap smears), HPV testing with polymerase chain reaction (PCR) testing, and visual inspection with acetic acid (VIA). Of these, the WHO generally recommends HPV-based screening every 5–10 years starting at age 30 years, based on the objectivity, reliability, and cost-effectiveness of the test.^29^ Research suggests that HPV self-swab collection is feasible and acceptable in Indigenous Guatemalan communities.^6^^,^^30^ After HPV testing, follow-up diagnostic processes also vary, but a see-and-treat model using VIA and same-day cryotherapy or thermocoagulation has proven acceptable, scalable, and cost-effective in LMICs, including in neighboring El Salvador.^31^^–^^37^ Follow-up after a diagnosis of cervical cancer depends on a multitude of factors and can be quite complex, underlining the importance of early screening and detection.

Approaches to enrollment and follow-up with individuals in cervical cancer screening and treatment programs vary by context. Screening approaches in the Global North often use technologies based on EHRs for targeting, outreach, screening, and follow-up.^38^^–^^42^ EHRs are less widespread in the Global South, especially in rural and historically marginalized communities, so these technological best practices are rare in these settings. Several cervical cancer interventions globally have used mHealth for educational and recruitment purposes, such as appointment reminders.^43^^–^^45^ However, we have not found reports of provider-facing mHealth interventions in the Global South or mHealth programs that support follow-up care.

Guatemalan national health care has been weakly funded and inequitably distributed, especially among Indigenous communities still reeling from the effects of a genocide that ended in the mid-1990s.^46^^–^^53^ This history, and ongoing inequities leave Guatemalans suffering from preventable illness and death, including from cervical cancer.^46^^,^^54^^,^^55^ Indigenous communities particularly lack reliable access to high-quality care, including in women’s health.^56^^–^^58^

We aimed to scale a culturally appropriate and accessible cervical cancer screening and treatment program in San Lucas Tolimán (SLT), a rural municipality, centering community health workers (CHWs) and community outreach. We describe the entire process, from initial assessment through program design and implementation.

## METHODS

SLT, located in the southwestern highlands of Guatemala, shows the marks of 500 years of colonization and disenfranchisement. Ongoing, long-term structural precarity leaves people at high risk for illness with few health care options. Within this rural municipality in the department of Sololá, formal employment is relatively rare. Most residents live day-to-day, often on less than US$2; community members cite cost and travel as common reasons for forgoing medical care.^59^ Additionally, community members here and elsewhere report distrust of medical systems that have historically provided substandard care, particularly to Indigenous women.^16^^,^^60^^–^^63^

Around 30,000 primarily Kaqchikel Maya Indigenous Guatemalans live in the town of SLT and 19 surrounding small villages, most of which fall within the SLT municipality.^64^ SLT has a small but longstanding rural health care program coordinated through a local nongovernmental organization (NGO) called Misión San Lucas.

Our team, which included women’s health providers (family medicine and obstetrics/gynecology) from North, Central, and South America and researchers and students from academic institutions in the United States, has a longstanding partnership with a team of CHWs in SLT. These CHWs, all of whom speak Spanish and some of whom also speak Kaqchikel, have previously worked with a mobile data collection platform called CommCare on ongoing mHealth-supported nutrition^65^^,^^66^ and diabetes^67^ programs. Our team first came together in early 2019, following a request from CHWs themselves for more information about women’s health and the possibility of a related intervention. Working directly with CHWs, we conducted a women’s health community needs assessment in 2019.^59^

We then conducted a pilot implementation screening and treatment program in SLT. Community education, distribution and collection of self-swabbing HPV kits, results, follow-up care, and referrals were coordinated through the SLT CHW program. Costs have varied from year to year, but annual overall costs have averaged around US$10,000.

### Baseline Community Needs Assessment

The specific methods of our 2019 community needs assessment have been described elsewhere.^59^ The survey was codesigned with CHWs. To summarize, we surveyed 61 women aged 18–75 years in Spanish or Kaqchikel about a variety of health topics using a mixed-methods approach and incorporated focus groups and key informant interviews with health care workers, as well as participant observation across health care and community settings. Supplement 1 lists the questions that pertained to cervical cancer, which we aimed to ask women aged 21–60 years. Although 61 women completed any part of the survey, the number of responses to specific questions varied. We used ExpressScribe (NCH Software, version 11.10, 2019) and NVivo (version 12, 2018) for qualitative analysis and Microsoft Excel (Microsoft Corp, version 16, 2021) and R (version 2023.06.1+524) for quantitative analysis.

### CommCare Data Collection and Tracking

Based on the results of the community needs assessment, we began to develop a cervical cancer screening program. Our team designed a mobile health application using the CommCare platform (version 2.53.1, 2023), which operates on Android devices, for use by the CHWs in recruitment, enrollment, tracking, and follow-up. Supplement 2 shows screenshots of basic enrollment within this app. The application also has a reporting module in Microsoft Excel (version 16.90, 2024) that CHWs use for generating metrics and data visualization.

### Community Health Worker and Physician Training

Before enrolling women in the program, we trained CHWs in Spanish on basic cervical cancer pathophysiology, self-kit use, and study protocols (training materials in Supplement 3). CHWs also learned to use the CommCare application and practiced using straightforward example scenarios. This 4–8 hour long training prepared CHWs to provide culturally sensitive education and assess women’s eligibility for screening.

Based on the educational materials, CHW coordinators designed a flipchart covering the basics of anatomy, cervical cancer, and HPV transmission. They also designed a second flipchart with specific instructions on the HPV self-swab based on instructions from the swab manufacturer (Supplement 4). The flipcharts mostly contained images with any text in Spanish. CHW coordinators used these flipcharts for community education before enrollment, choosing to provide this education in Spanish, Kaqchikel, or both, based on the audience. We also developed video guidance for self-kit use in Spanish and Kaqchikel.

The project trained 2 SLT physicians—general practitioners employed at the hospital that supervised the CHW program—on cervical cancer screening, diagnosis, and follow-up. Two groups led these trainings, including 2 ob/gyn physicians and 1 family medicine nurse practitioner, all with global health and cervical cancer treatment experience. Training entailed multiple synchronous, virtual meetings to review VIA approaches and then a total of 4 days in-person working collaboratively on VIA, cryotherapy, and thermocoagulation.

### Study Population and Recruitment

We sought to screen women aged 30–49 years (WHO Guidelines 2021) within the 19 rural villages and the town of San Lucas within the municipality of SLT. Women speak Spanish and/or Kaqchikel. Women were excluded if they were pregnant, had a known cervical cancer diagnosis, or were experiencing postcoital bleeding. We prioritized women for screening if they were in the age range and had not had a Pap smear with a conclusive negative result in the last 3 years. We did not screen women for HIV or include an HIV-specific arm in our protocol. This was due to the low prevalence of HIV in this community, estimated at 0.2% in Guatemala and thought to be lower in Indigenous communities.^68^ Women menstruating when they received the kit were counseled to wait until after they stopped bleeding to complete the self-swab. Women menstruating at the time of the VIA exam were examined according to provider discretion.

Women were recruited using purposive sampling based on CHW community outreach. CHWs and CHW coordinators asked women if they were within the age group and if they might be interested in attending a women’s health educational chat with their local CHW.

To provide physicians with adequate training while visiting physicians were present, 2 groups of women received VIA: a group that had already tested positive for HPV, which followed the overall study protocol, and a group that had never been screened. After physician training ended, the pilot program proceeded to screen using HPV and use VIA for follow-up only for women positive for HPV.

### Self-Collected Samples

CHW coordinators led community education with interested women. Women who enrolled then received a self-swab kit, the Evalyn Brush by Rover (Supplement 4). Women who agreed to participate explicitly agreed to perform their swabs themselves. Most women chose to collect their self-swab at home, while some women requested to complete the self-swab in the CHW’s home or a community restroom with supervision. The community’s CHW then collected tests from participating women, aiming to collate swabs within 1 week of swab collection. Swabs could be stored at room temperature for up to 90 days before being sent to the lab and did not require refrigeration or additional packaging when being sent to the lab, which was in Guatemala City. CHW coordinators aggregated and labeled tests in batches to send to our partner lab packed in a lab-approved box within 1 month of sample collection, and the lab completed PCR testing. Results, including subtypes (16, 18, and/or non-16/18), were returned within 10 days. CHWs then communicated results with women through in-person visits. CHWs logged every step of this process in CommCare.

### Further Screening and Treatment Measures

If a woman tested positive for HPV, regardless of subtype, CHWs invited her for further screening at the local NGO hospital. Physicians then performed VIA for diagnosis with follow-up same-day treatment using cryotherapy or thermocoagulation as necessary. Women were eligible for same-day treatment if their entire lesion and squamocolumnar junction were visible with no extension into the endocervix or onto the vaginal wall, their lesion was small enough to be covered by the cryoprobe, and their lesion covered less than three-quarters of the ectocervix. They were not eligible if providers found evidence or suspicion of invasive disease or adenocarcinoma in situ; if the lesion extended beyond the cryoprobe edge; or if the woman was pregnant, actively menstruating, or had untreated pelvic inflammatory disease.

Women with lesions suspicious for precancer or cancer who were not eligible for same-day treatment were referred to visiting surgical groups, with client education provided both at the time of VIA and through subsequent CHW home visits to help address any of the woman’s concerns. Within 6 months of VIA, these women received loop electrosurgical excision procedure (LEEP) or cervical conization per the surgeon’s clinical judgment. Biopsies from these procedures were sent to pathology.

Women who were not initially eligible for localized treatment or received a pathological diagnosis of invasive cancer were referred to the national cancer hospital (Supplement 5 details the screening protocol). A combination of study and NGO funds covered all treatment and transport to treatment at no cost to women or their families.

Women who tested negative for HPV were due for HPV retesting in 5 years unless they aged out. Women who were positive for HPV with negative VIA get retested for HPV in 24 months (Supplement 5).

### Statistical Analyses

HPV-positive rates were compared with national and subpopulation-level HPV rates to determine the significance of HPV positivity in SLT. The significance of HPV+ rates was determined using 2-tailed proportion tests. Statistical analyses were conducted using the programming language R packages readxl and dplyr.

### Ethical Approval

The community needs assessment element of this study was deemed exempt by the University of Wisconsin Institutional Review Board. The implementation of cervical cancer screening was deemed to be a quality improvement/program evaluation project not requiring further Institutional Review Board review. We worked directly with the health care committee of the local NGO to ensure ongoing local ethics oversight.

## RESULTS

### Interest in Cervical Cancer Screening

In our initial community needs assessment, questions about cervical cancer prompted interest and discussion. Quantitatively, 90% (54/60) of women had heard of cervical cancer. Of the 60 initial women respondents, 58 women completed the rest of the survey about cervical cancer. About half (51.7%; 30/58) had previously undergone a Pap smear. While some had had more than 1 screening, no one interviewed had had regular screening per WHO recommendations. Of 27 women who shared the outcome of their most recent Pap smear, 1 reported a diagnosis of precancer (cervical intraepithelial neoplasia 1), and 21 (77.7%) reported their result was “normal,” “negative,” or “good.” Five women (18.5%) said they were diagnosed with an “infection,” rather than a positive or negative result, from their Pap smear; none of the 5 ever received follow-up. The vast majority (96.5%, 56/58) of interviewees expressed interest in CHW-led discussions of cervical cancer screening.

When women were asked about screening modality, specifically whether they would prefer to take a sample themselves versus a provider performing a pelvic exam to collect a sample, 74.5% of women (38/51) said they would prefer a provider conduct the test. Qualitatively, in relation to the preferred screening modality, many women tempered their response to the prior either/or quantitative question by saying they would prefer to defer to provider expertise and whichever was the “better test.” Women reported reluctance to receive pelvic exams for cervical cancer screening. Women cited privacy concerns, particularly if they would be seen by male physicians.

Six women said they had not received a Pap smear due to “fear,” particularly based on stories they had heard from others about painful exams. Some worried that the exam would make them sick. Several women mentioned “vergüenza” (meaning “shame” or “embarrassment”) when talking about the exam. Three women reported not having attended screening because they didn’t see any need for screening if they were not having symptoms or did not feel sick enough to go. However, the most common reason cited for not having a Pap smear was the lack of availability of affordable services in their communities. Many people reported knowing someone or knowing of someone who had died of cervical cancer. Women also shared stories where community members had received cervical cancer screening and a diagnosis of “some sort of cancer” but were left to die at home.

In focus group discussions, key informant interviews, and based on participant observation with health care providers, cervical cancer was perceived as a “low-hanging fruit” for intervention. Providers reinforced themes from community surveys, including lack of follow-up care even where screening was available and a perception that women were embarrassed to receive pelvic exams, particularly from male physicians.

Providers and participant observations from clinical encounters also corroborated the quantitative findings. Particularly, health care providers and women agreed that Pap smears often came back from cytology with a nonspecific diagnosis of “inflammation.” Women were often told to treat this inflammation (often assumed to be bacterial vaginosis, for which women were prescribed ovulos [vaginal metronidazole suppositories] that required out-of-pocket payment, and then return for a repeat Pap smear 6 months later. No women who received this diagnosis reported having a follow-up Pap smear. Providers’ perception of inadequate lab and follow-up infrastructure for cervical cytology prompted our team’s proposal to provide HPV testing with follow-up VIA.

### Training

In preparation for teaching about cervical cancer, HPV, and self-swab collection, CHWs took pre- and post-tests to assess comprehension and identify additional training needs. The average pre-training score was 60.8% (range 0%–80%). On the post-test, CHWs averaged 90.0% (range 60%–100%). Qualitatively, CHWs reported being able to comfortably discuss these topics with their neighbors despite taboos.

### Enrollment

As of November 2023, 256 women had received education through a community-based educational chat, and 230 of these women were eligible to participate. During the study period, 215 received screening. Of these, 67 women were recruited directly to VIA exams, and the others (n=162) received HPV tests. Table 1 shows the demographic characteristics of the women in the program, and the Figure shows the screening and treatment program process. While we did not formally collect discrete feedback from women, we heard from CHWs that women overall liked being able to conduct the self-swab and appreciated the chance to do HPV screening without having to go to a clinic or undergo a pelvic exam. CHWs also reported that some women, despite receiving community education, preferred not to conduct screening.

**TABLE 1.** Demographic Characteristics of Women Surveyed for Eligibility in a Cervical Cancer Screening Pilot Program, San Lucas Tolimán, Guatemala

	**No. (%)**		
	**Total Surveyed**	**HPV Test**	**VIA Exam**
Total	256 (100)	132 (51.56)	67 (26.17)
Age, years			
Younger than 18	0	0	0
18–29	7 (2.73)	0	1 (1.49)
30–49	245 (95.70)	132 (100)	64 (95.52)
50 or older	4 (1.56)	0	2 (2.98)
Occupation			
Homemaker	230 (89.84)	115 (87.12)	63 (94.02)
Seasonal worker	3 (1.17)	2 (1.15)	2 (2.98)
Full-time worker	0	0	0
Other	4 (1.56)	1 (1.00)	1 (1.49)
N/A	19 (7.42)	14 (10.61)	1 (1.49)
Marital status			
Married	126 (49.22)	54 (40.91)	36 (53.73)
Single	18 (7.03)	11 (8.33)	8 (11.94)
Together, not married	89 (34.77)	52 (39.40)	21 (31.34)
Widowed	4 (1.56)	1 (1.00)	1 (1.49)
N/A	19 (7.42)	14 (10.61)	1 (1.49)
History of Pap smear			
Yes	107 (41.80)	46 (34.85)	53 (79.10)
No	149 (58.20)	86 (65.16)	14 (20.89)

Abbreviations: HPV, human papillomavirus; N/A, not available; VIA, visual inspection with acetic acid.

**FIGURE:**
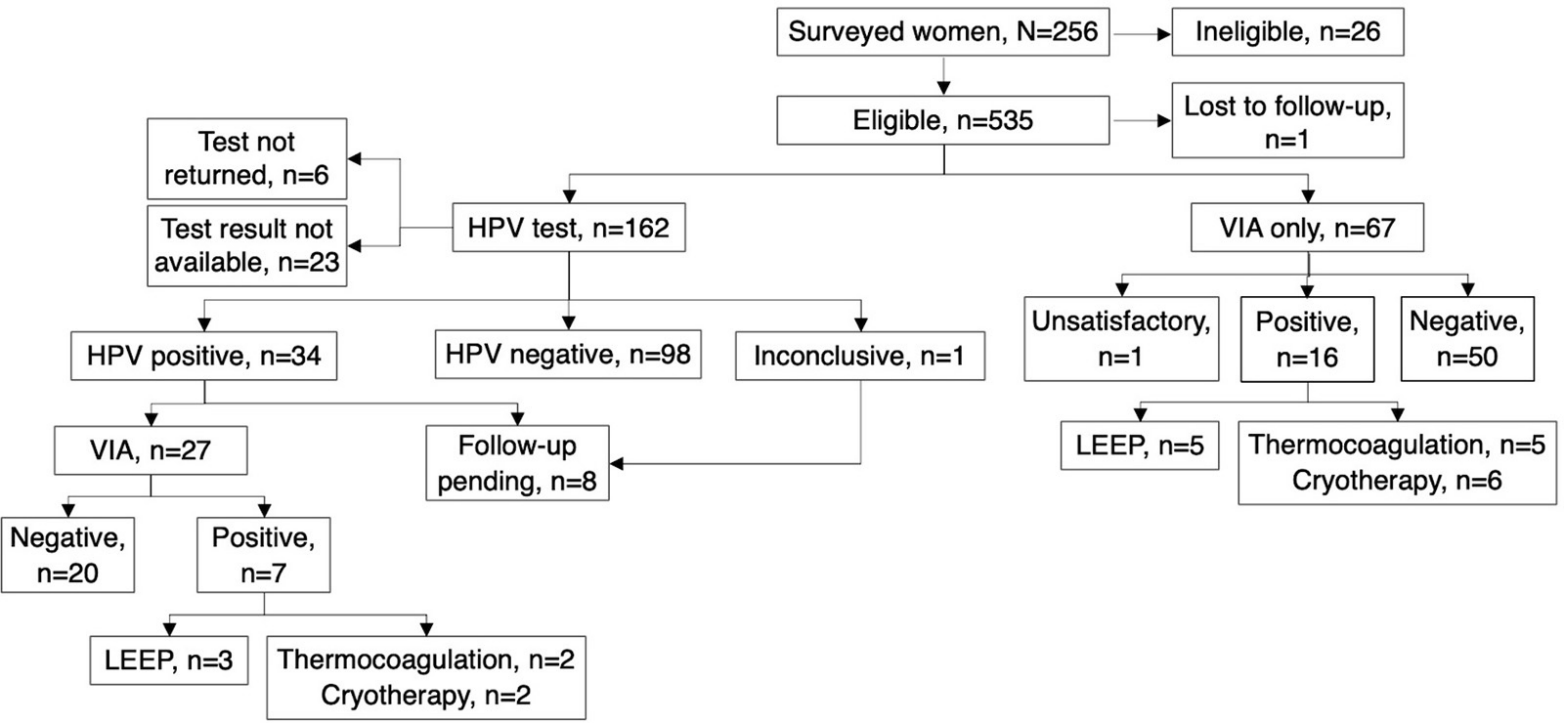
Cervical Cancer Screening and Treatment Pilot Program Process for Participants, San Lucas Tolimán, Guatemala Abbreviations: HPV, human papillomavirus; LEEP, loop electrosurgical excision procedure; VIA, visual inspection with acetic acid.

### Previous Screening

We asked women at enrollment if and when they had any sort of cervical cancer screening. Of 256 women who received educational chats, 107 (41.79%) said they had ever received a Pap smear. Compared to rates in other Indigenous communities in the Highlands,^12^ this rate is not statistically different (*P*=.63).

### Human Papillomavirus Test Results

CHWs distributed HPV self-swab kits to 162 women. Of the 132 women with conclusive results, 34 were positive for high-risk HPV (25.76% prevalence). Observed HPV subtypes were: genotype 16, 11.76% (4/34); genotype 18, 8.82% (3/34); non-16/18, 73.53% (25/34); and genotype 16 with another non-18 genotype, 5.58% (2/34).

### Visual Inspection With Acetic Acid Exam Results

A total of 94 women received VIA exams (Table 2), of which 27 were following up after a positive HPV test and 67 were recruited directly to VIA for training purposes. Seven women with HPV did not attend follow-up clinics; some of these women had follow-up pending at the time data were collected, and other women decided to opt out of further care.

**TABLE 2.** VIA Exam Results, San Lucas Tolimán, Guatemala

	**No. (%)**			
		**Results**		
	**Total Who Had VIA Exam**	**Positive**	**Negative**	**Unsatisfactory**
VIA only	67 (71.28)	16 (23.88)	50 (74.63)	1 (1.49)
HPV positive and VIA	27 (28.72)	7 (25.93)	20 (74.07)	0
Total	94 (100)	23 (24.47)	70 (74.47)	1 (1.06)

Abbreviations: HPV, human papillomavirus; VIA, visual inspection with acetic acid.

Of the 94 women who received VIA exams, 23 had positive exams (24.47%), 70 had negative (74.47%), and 1 had a test that was unsatisfactory (1.06%). In VIAs among women not previously screened for HPV, 23.88% (16/67) were positive. Conversely, VIA positivity among women who were positive for HPV was 25.93% (7/27).

Women with positive VIA exams, that is, concerning lesions, received subsequent cryotherapy (n=8), thermocoagulation (n=7), or LEEP (n=8) (Table 3).

**TABLE 3.** Same-Day Treatment for Women Who Had Positive VIA Exam Results, San Lucas Tolimán, Guatemala

	**No. (%)**			
	**Total Who Received Treatment**	**Thermocoagulation**	**Cryotherapy**	**LEEP**
VIA only	16 (69.57)	5 (31.25)	6 (37.50)	5 (31.25)
HPV positive and VIA	7 (30.43)	2 (28.57)	2 (28.57)	3 (42.86)
Total	23 (100)	7 (30.43)	8 (34.78)	8 (34.78)

Abbreviations: HPV, human papillomavirus; LEEP, loop electrosurgical excision procedure; VIA, visual inspection with acetic acid.

Pathological diagnoses (Table 4) ranged from inflammation to cervical intraepithelial neoplasia I and II, with 1 diagnosis of invasive squamous cell carcinoma. Women were scheduled for follow-up examinations within the CommCare app after their excisional procedures. Two women had heavy bleeding and were seen for this complication in the days following their procedure; bleeding control was achieved in both cases. Women continued to receive visits from CHWs and were encouraged to contact them at any time with concerns. CHWs reached out to study physicians as needed. Of note, the woman (n=1) who received an advanced cervical cancer diagnosis has thus far refused care at the national hospital.

**TABLE 4.** Biopsy Results After Treatment, San Lucas Tolimán, Guatemala

**Excisional Modality**	**Final Diagnosis**	**No.**
LEEP	Cervicitis/inflammation	3
LEEP	Polyp with cervicitis	1
LEEP	CIN I	2
LEEP	CIN II	1
Cold knife conization	Invasive cancer	1

Abbreviations: CIN, cervical intraepithelial neoplasia; LEEP, loop electrosurgical excision procedure.

### Human Papillomavirus Rates in Local and National Context

We performed a 2-tailed proportion test to compare HPV rates in SLT to positivity rates at the national level,^5^ in Escuintla,^69^ and in Santiago Atitlán.^18^ We found a statistically significant difference in the sample HPV positivity rates in SLT compared to the national average HPV positivity rates (12.4%, *P*<.01). The HPV rates we found were also higher than other communities in the region that have been studied. SLT had higher HPV positivity rates than Escuintla to the south (21.6%; *P*=.29), though this difference did not reach statistical significance. However, compared to rates in Santiago Atitlán to the west (17.4%), SLT HPV positivity rates were significantly higher (*P*=.02).

## DISCUSSION

We designed and implemented a cervical cancer screening and treatment program in rural Guatemala. We aimed to combine existing best practices and attempt to provide high-quality care at no cost to women. We incorporated best practices from Guatemala and elsewhere, including working collaboratively with CHWs to provide education in sensitive and thoughtful ways;^13^^,^^25^^,^^27^^,^^70^ sourcing home tests to address women’s privacy concerns;^6^^,^^18^^,^^30^ using high-quality PCR-based HPV testing to target follow-up;^71^^,^^72^ training local physicians in VIA^26^ with same-day cryotherapy^32^ and thermocoagulation;^31^ and integrating visiting specialists only with local coordination and follow-up.^60^^,^^73^ We also hope to coordinate with national cancer resources for women with advanced disease.^16^^,^^54^ Although each of these components has been used by other researchers, we are one of a few projects in Guatemala that have, to date, implemented all these components together in a comprehensive cervical cancer program. Our goal throughout has been to provide locally responsive, community-based, respectful, and cost-free care for women.

This study further confirms prior research as well as our study’s initial findings that women in low-income settings are interested in cervical cancer screening^4^^,^^74^ but are wary about undergoing pelvic exams.^4^^,^^12^^,^^27^ The survey results also reinforce previous literature that screening methods at the time of our study in Guatemala did not adequately reach at-risk populations.^9^^,^^12^^,^^16^^,^^24^ Women and health care providers’ emphasis on test quality (belying quantitative preference for provider-collected tests) led us to pursue HPV testing rather than Pap smears or VIA as the first screening. This investment was borne out by the perspectives women shared with CHWs; though some opted out of the screening, many appreciated the chance to conduct self-swabs privately in their own homes without provider-led pelvic exams.

Our quantitative results supplement the limited existing data on HPV and cervical cancer in Guatemala, specifically among Indigenous Guatemalan women. Our findings corroborated our initial community needs assessment findings that only around half of women had ever had cervical cancer screening. Our observed Pap smear rates were statistically similar to nationwide rates^18^ and among Indigenous women in highland communities.^12^ Our study’s low lifetime screening rates in a municipality that, to our knowledge, had not previously been surveyed beyond our study team reinforce the need for community-based programs integrating screening, treatment, and follow-up.

Our study’s HPV positivity rate, significantly higher than what was found in previous studies in other sites, may suggest more widespread HPV positivity in rural Indigenous Guatemalan communities. We are interested in the geographical distribution of HPV positivity and propose future work studying whether certain areas have a greater need for HPV screening and prevention than others in Guatemala.

Our program also showed the preliminary success of our CommCare-based mHealth tracking and follow-up program. Other projects have tested mHealth outreach via short message service (SMS) to women for cancer prevention and treatment^43^^,^^44^ or have incorporated EHRs within government screening programs, used by doctors, nurses, and technical assistants.^45^ To our knowledge, this is the first use of a CHW-facing mHealth intervention in the Global South for a cancer screening program. The team members who collaborated to develop these tools all either worked full-time in SLT or had spent time there learning about the CHW program writ large and the cervical cancer project specifically, allowing the tool to be tailored specifically to the program’s needs. Although our analyses did not directly evaluate the use of this tool, our research team and collaborators found CommCare to be useful and conducive to long-term follow-up for cervical cancer screening. We continue to make improvements to this application as needed and can share CommCare code and best practices with other projects seeking similar approaches.

Our screening program expansion highlighted ongoing fragmentation and sustainability concerns. We lost women to follow-up at every step, despite house-to-house outreach. Ongoing work by team members aims to analyze care refusal and improve outreach. Our providers’ lack of confidence with pathological diagnoses has led us to seek other routes to send biopsies, and we continue to work with American and Guatemalan pathologists to try to develop long-term approaches. As we are able to identify women with advanced cancer, we aim to strengthen ties to the national hospital using an accompaniment model that has been successful in providing cervical cancer care in similar communities^54^ but is as of yet untested in SLT. We are also considering expanding the age of tested women in rural areas to 65 years, a request from our CHW partners to provide screening for a group of women who qualitatively have expressed concern about cervical cancer. We are piloting SMS reminders in another study with the same group of CHWs, which could serve as an additional tool to reach women.^75^ We hope these quality improvements can help address women’s hesitancy to get cervical cancer care.^4^^,^^12^^,^^16^^,^^76^

Findings from our and others’ implementation projects demonstrate that it is feasible and cost-effective to screen for, treat, and eventually eliminate cervical cancer in Guatemala.^5^^,^^6^^,^^12^^,^^13^^,^^18^

### Strengths and Limitations

This project benefits from strong local partnerships and dedication to equitable care. Because of these priorities, we based our approach on 1 community needs assessment; we did not, therefore, capture the larger screening, vaccination, and treatment framework in the municipality, department, or country for cervical cancer. After we finished data collection for this project, some of our team members began a subsequent project working directly with local and national Ministry of Health experts to collaborate on ongoing, robust cervical cancer prevention within the public health system. The Ministry of Health has previously used NGO pilot data in its broader scale-up of prior cervical cancer screening projects, specifically in using VIA as a first screening.^77^ Therefore, we are hopeful that our small study can contribute to ongoing screening capacity scale-up countrywide.

Our still-small sample size limits our ability to generalize our data to the larger population. For example, we would like to be able to use HPV vaccination rates to determine best practices in cervical cancer screening, but very few women reported knowing about, let alone receiving, HPV vaccinations. The government HPV vaccination program to vaccinate girls started in 2018, first with girls aged 10–11 years and then expanding to age 10–14 years.^10^ Although some women reported receiving HPV vaccination, they did not have documentation. Local study team members doubted that the vaccines women (aged 30–49 years) remember receiving were truly for HPV but had no way to confirm this. This is an example of how infrastructure limitations that can routinely prevent women from getting needed essential health services can also hinder data collection and quality improvement.

A key limitation of our project was its isolation within 1 NGO. An ongoing debate in global health in Guatemala and beyond challenges global health funders, especially from the Global North, to focus program and infrastructure support on national health programs rather than developing so-called parallel systems through NGOs.^55^^,^^78^^,^^79^ Parallel systems, scholars and practitioners caution, continue to weaken the mandate on governments and international funders to strengthen public health, duplicate effort, and perpetuate neocolonialism.^80^^,^^81^ Furthermore, when funding (like that which supported this project initially) is linked to research outcomes, scholars can perpetuate real harm in communities that have long been experimented upon, coerced into being obligatory study subjects instead of autonomous people, often because they have no other route to care.^82^^,^^83^ Our team consisted of local as well as international scholars and providers, and we hope that we have not perpetuated the worst of these patterns. With this in mind, we structured the program to be CHW-run and managed, providing culturally and linguistically appropriate care from local community members and providers. Our team members are pursuing follow-up implementation work that collaborates directly with public health leaders to attempt to bring the lessons of this small pilot to the larger Guatemalan health system.

One of the more difficult parts of this project, which we did not have a chance to discuss in this article, was the process of using funding from a U.S.-based university to pay for HPV tests in Guatemala. Our months-long struggle to be able to pay our partners and the attendant delays in care again raised concerns about sustainability, equity, and fragmentation. We were able to secure long-term funding for cervical cancer screening, treatment, and follow-up, including transport and cancer treatment when needed, through the NGO we work with, but this approach is not scalable to communities that do not have this type of NGO support. We hope to further explore ways that partnerships can be supported (or hindered) by various funding mechanisms.

Our project benefited from local flexibility and creative approaches to develop training and scale-up. Collaborating with local experts strengthened our study team’s deep connections in the community. Definitionally, then, our study is not blinded nor representative in its sampling. We never intended for this study to be generalizable; instead, we hope it serves as a proof of concept. Additionally, we started very small and, therefore, at the time of writing still have only tentative links with national cancer institutes and follow-up care. This must improve. But we hope the roadmap we have provided, including detailing where we struggled, can help future scholars and practitioners.

## CONCLUSIONS

We discussed outcomes of an ongoing project providing screening, treatment, and follow-up for HPV and cervical cancer in 1 majority Indigenous municipality in Guatemala. We found higher HPV rates than national averages and comparable or higher rates to nearby areas of Guatemala. Our experience showed that there is a high demand for HPV testing and follow-up and that rural and Indigenous Guatemalan women still lack reliable access to HPV screening and cervical cancer follow-up. We found that providing self-swab kits was feasible and accepted by community members. CHWs successfully used an mHealth application that evolved as the program grew, which proved a useful way to enroll women and track follow-up. In addition to the benefit to the local community, our project has global ramifications, as many countries share Guatemala’s high cervical cancer rates. We welcome the opportunity to collaborate with other programs, including by sharing our mHealth application code, to support HPV self-swab collection and follow-up treatment to address cervical cancer among communities in need worldwide.

## Notes

**Peer Reviewed**
